# Novel Therapeutic Approaches in Treatment of Acute-on-Chronic Liver Failure

**DOI:** 10.1055/s-0043-1776773

**Published:** 2023-12-15

**Authors:** MohammadMahdi Saeidinejad, Ahmed Elshabrawi, Supachaya Sriphoosanaphan, Fausto Andreola, Gautam Mehta, Banwari Agarwal, Rajiv Jalan

**Affiliations:** 1 Liver Failure Group, Department of Medicine, Institute for Liver and Digestive Health, University College London, London, United Kingdom; 2 Intensive Care Unit, Endemic Hepatology and Gastroenterology Department, Mansoura University, Mansoura, Egypt; 3 Division of Gastroenterology, Department of Medicine, Faculty of Medicine, Chulalongkorn University and King Chulalongkorn Memorial Hospital, Thai Red Cross Society, Bangkok; 4 Intensive Care Unit, Royal Free Hospital, London, United Kingdom; 5 Hepatology Department, Royal Free Hospital, London, United Kingdom; 6 European Foundation for the Study of Chronic Liver Failure, Barcelona, Spain

**Keywords:** cirrhosis, ACLF, Inflammation

## Abstract

Acute-on-chronic liver failure (ACLF), a clinical syndrome that can develop at any stage in the progression of cirrhotic liver disease, is characterized by an acute decompensation in liver function with associated multiorgan failure and high short-term mortality. Current evidence points to ACLF being reversible, particularly in those at the lower end of the severity spectrum. However, there are no specific treatments for ACLF, and overall outcomes remain poor. Expedited liver transplantation as a treatment option is limited by organ shortage and a lack of priority allocation for this indication. Other options are therefore urgently needed, and our improved understanding of the condition has led to significant efforts to develop novel therapies. In conclusion, this review aims to summarize the current understanding of the pathophysiological processes involved in the onset, progression, and recovery of ACLF and discuss novel therapies under development.

## Graphical abstract

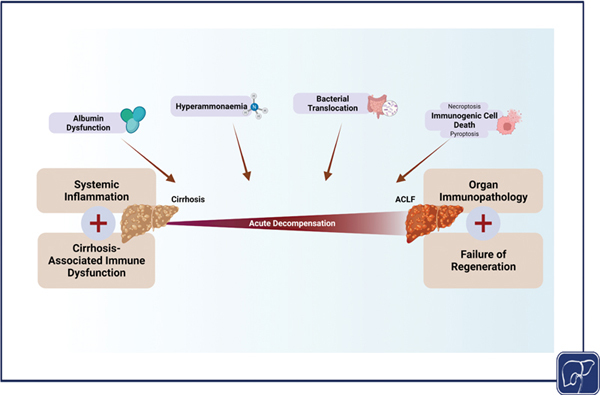

Our understanding of liver cirrhosis, its progression, prognosis, and pathophysiological features, has improved considerably over the recent decades. The traditional dichotomous view of compensated and decompensated stages has evolved to reflect the more nuanced and variable clinical course that follows acute decompensation (AD) in hospitalized patients. Two distinct subtypes are identified; acute-on-chronic liver failure (ACLF) is the most severe expression of AD and is characterized by intense systemic inflammatory response, systemic organ failures, and high short-term mortality. 1 ACLF is common and is present in approximately 30% of the patients with cirrhosis at the time of hospital admission with a further 10% developing ACLF during hospital stay (pre-ACLF). 2 The high short-term mortality has been confirmed in several cohorts from across the world regardless of the definition used. 2 3 4 5 6 The 28-day mortality in the CANONIC study, the first major prospective large-scale study of all ADs across multiple centers in Europe, was 32.8% in patients with ACLF compared with less than 2% in those without ACLF. Additionally, the mortality correlates with the number of organ failures and ACLF grade (23% for ACLF grades-1 to 77% for grade-3, 28-day mortality). 2 7 8 These data have since been validated in multiple studies. 1 Three distinct clinical courses have been observed in patients with no ACLF (AD); these patients may develop stable decompensation, unstable decompensation, or pre-ACLF ( Fig. 1 ). These are pathophysiologically and clinically distinct subgroups with variable levels of systemic inflammation, surrogates of portal hypertension, organ dysfunction/failure, and outcomes. The natural history of pre-ACLF differs significantly from other acutely decompensated states with evidence of more severe systemic inflammation (higher C-reactive protein [CRP] and white blood cell count [WBC]), ascites, and presence of organ dysfunction (liver failure/dysfunction, kidney and coagulation dysfunctions) making them prone to develop ACLF. 9 10

**Fig. 1:**
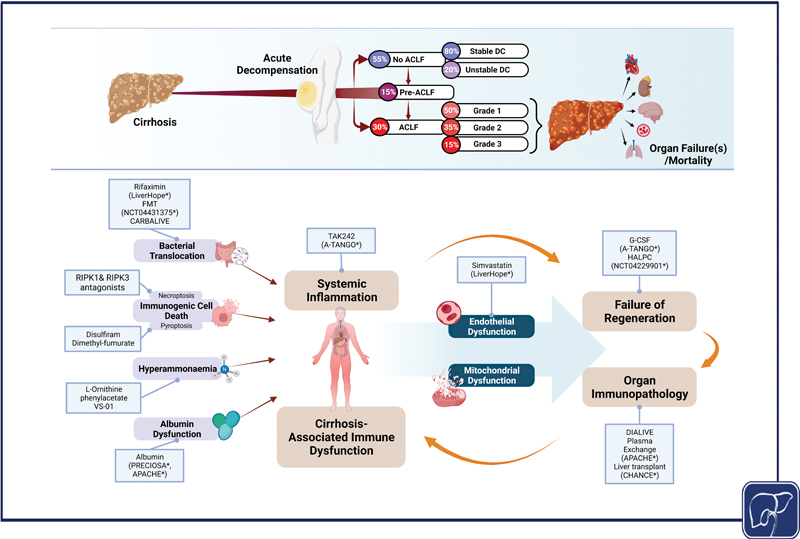
The overview of the disease progression model ( *top* ) and the pathogenesis of ACLF with the investigated treatment targets ( *bottom* ). The relevant studies to each treatment have been marked with * and placed inside brackets. ACLF, acute-on-chronic liver failure; FMT, fecal microbiota transplant; G-CSF, granulocyte–colony stimulating factor; HALPC, human allogenic liver-derived progenitor cells; RIPK, receptor-interacting protein kinase.

Different operating models of defining ACLF, assessing the severity and prognostication, have been proposed by international liver associations ( Table 1 ); the European and North American definitions recognize the importance of organ failures, 2 11 12 whereas the Asian Pacific Association for the Study of the Liver (APASL) definition excludes previous episodes of decompensation and extrahepatic precipitating events. 10 The European Association for the Study of the Liver Chronic Liver Failure Consortium (EASL CLIF-C) model is the most widely validated model for the diagnosis and prognosis of ACLF, 1 and will be used in this review unless specified otherwise.

**Table 1:** Main characteristics of acute-on-chronic liver failure as described by international groups

	European Association for the Study of the Liver-Chronic Liver Failure (EASL-CLIF) Consortium	North American Consortium for the Study of End-Stage Liver Disease (NACSELD)	Asian Pacific Association for the Study of the Liver (APASL)-ACLF Research Consortium (AARC)
Articles reporting the definition	The CANONIC study in 1,343 patients with cirrhosis nonelectively admitted to the hospital Eligible patients: ● Patients with acute decompensation, with/without prior episodes of decompensation	A prospective study in cirrhosis patients with acute decompensation due to infection Eligible patients: ● Patients with acute decompensation, with/without prior episodes of decompensation	A consensus from experts and the AARC database Eligible patients: ● Patients with first episode of acute decompensation in compensated cirrhosis or non-cirrhotic liver disease
Precipitating events	Intrahepatic and extrahepatic	Intrahepatic and extrahepatic	Intrahepatic (excludes infection)
Definition of organ failure	Six organs assessed with CLIF-OF score. Cut-offs defined agnostically	Four organs defined by need for organ support (brain, kidney, respiration, circulation)	Need for organ failure not relevant

Abbreviations: ACLF, Acute-on-chronic liver failure; OF, organ failure.

The current standard of care (SOC) for managing ACLF follow the PIRO principle, which encompass the treatment of underlying liver disease (Predisposition), the precipitating events (Injury), inflammation and infection (Response), and provision of support for failing organs (Organ). 13 There are no specific approved treatments for ACLF per se. With SOC, patients either continue to deteriorate, improve, or stay unchanged over 3 to 7 days displaying a dynamic clinical course. 7 The final severity grade informs the onward trajectory of outcomes, irrespective of the severity at presentation. Resolution of ACLF is observed in approximately 54, 34, and 16% patients with ACLF grades 1, 2, and 3, respectively. An understanding of the natural history of ACLF allows determination of potential futility of ongoing supportive care or selection and prioritization for liver transplantation (LTx). 2 LTx is the only rescue option for patients unresponsive to standard care and has been shown to improve outcomes with 1-year survival rates exceeding more than 80% 14 15 16 and long-term survival post-LTx similar to those transplanted with no ACLF. 17

These data highlight the potential for resolution of even the extrahepatic organ failures over time, with standard medical care or LTx, thus providing the clinical rationale that novel therapies for ACLF have the potential to impact on long-term outcomes since the syndrome shows a degree of reversibility. Hence, the surrogate endpoint and aim of any intervention may well be resolution of ACLF. Moreover, the clinical course provides the information on the timing of any novel therapeutic approach and defines the baseline for determining the potential effect size of any novel treatment to be tested.

Cirrhosis, AD, and ACLF fall within the same spectrum. While the therapeutic aims seemingly differ at each stage, the common goal is to address systemic inflammation and immune dysfunction, a shared theme across the spectrum, albeit with different grades of severity. Considering this, our review will cover preclinical and clinical therapeutic studies across this disease spectrum, which either directly treat ACLF or result in its prevention. However, our endeavor has been to focus mostly on direct therapies for ACLF.

## Pathophysiology of Acute-on-Chronic Liver Failure and Potential Therapeutic Targets

Novel approaches for the treatment of ACLF are underpinned by the changed pathophysiological paradigm of disease progression from traditionally held peripheral vasodilation and circulatory dysfunction hypothesis 18 19 to the recognition of the importance of systemic inflammation. According to the former hypothesis, end-organ dysfunction in patients is dictated predominantly by vascular dysfunction, peripheral vasodilation, portal hypertension, activation of neurohormonal systems, and organ hypoperfusion. 18 The current hypothesis of organ dysfunction ( Fig. 1 ) recognizes the importance of cirrhosis-associated immune dysfunction (CAID) 20 and the associated systemic inflammation 21 as the central pathogenetic mechanisms responsible for this syndrome. In this model, bacterial translocation, 22 immunogenic modes of cell death such as necroptosis and pyroptosis, 23 hyperammonemia, 24 and albumin dysfunction 25 are identified as important contributors to the development of CAID. This in combination with systemic inflammation and its downstream effects on endothelial dysfunction, 26 metabolic failure resulting from mitochondrial dysfunction, 27 and failure of regeneration culminate into organ immunopathology resulting in organ failure. 28 CAID promotes further translocation of pathogens and liver cell death with worsening immunopathology in a vicious cycle. Upstream events such as infections or sterile inflammation leading up to the development of CAID and systemic inflammation could be targeted to prevent the occurrence of ACLF and treat early ACLF.

In summary, current novel strategies are directed toward modifying factors at all stages of the disease process from predisposition, injury, response to organ dysfunction. 13 The preclinical and clinical studies targeting each of these pathways will be described below. Treatment of the underlying liver disease, management of the precipitating event, and intensive care support are reviewed elsewhere 29 and will not be described further.

## Preclinical and Clinical Status of Novel Therapies in Development

### Targeting Systemic Inflammation

Findings from the CANONIC study suggest that systemic inflammation is a characteristic feature in patients with ACLF 2 where higher levels of WBC and plasma CRP have been observed compared with those with AD without ACLF. 2 Elsewhere it was observed that the effectors of systemic inflammation, such as interleukin (IL)-6, IL-1β, and IL-8, are higher in patients with ACLF than those without. 30 Theoretically, targeting systemic inflammation should prevent or at least ameliorate the course of ACLF. Yet the situation is more complex due to many confounding factors. CAID and systemic inflammation are the main drivers of organ immunopathology and organ failure and attempts to suppress systemic inflammation can worsen CAID resulting in an increased risk of infection, a drawback that is well recognized in those treated with steroid for alcoholic hepatitis. 31 32

Targeting systemic inflammation through tumor necrosis factor α (TNFα) inhibition has been studied in patients with alcoholic hepatitis. Small preliminary studies of infliximab showed encouraging results 33 34 leading to a larger randomized placebo-controlled study of infliximab and prednisolone in those with severe alcoholic hepatitis. 35 Unfortunately, this had to be stopped early due to a significant increase in severe infections and nonsignificant increase in deaths in the infliximab cohort. Similar results were seen in a small pilot study of etanercept resulting in worse 6-month survival rate in the treatment group compared with placebo. 36 Similarly, inhibiting IL-1 had been shown to reduce inflammatory cell infiltrates and increase proliferation in a mouse model. 37 However, a randomized trial of anakinra in combination with pentoxifylline and zinc sulfate did not result in significant improvement in survival compared with steroid therapy. 38 The data suggest that adopted treatment methods should aim to target systemic inflammation through modulation of the immune system rather than a mere suppress or enhance approach. 39

Alcoholic hepatitis is an important precursor to ACLF and in the STOPAH trial 40 around 28% developed ACLF. This is remarkable considering that the study had excluded those with renal failure and inotropic requirement. Furthermore, in the Erasme Hospital cohort 41 48% patients with biopsy-proven severe alcoholic hepatitis were found to have ACLF. Therefore, therapeutics, which fail to show positive results in studies of patients with alcoholic hepatitis, are unlikely to be of benefit in ACLF.

### Targeting Pathogen-Associated Molecular Patterns

Pathogen-associated molecular patterns (PAMPs) are microbial molecular patterns detected by the innate immune system through specific pattern recognition receptors (PRRs). The result of this pairing is the stimulation of signaling pathways that lead to an inflammatory response, the purpose of which is to induce host resistance to pathogens. 42 For instance, the binding of lipopolysaccharide (LPS) to toll-like receptor 4 (TLR-4), present on the surface of many parenchymal and nonparenchymal cells, leads to the activation of key transcription factors, nuclear factor-κB, and interferon-regulatory factor, resulting in cytokine and interferon production. 20 This response is exaggerated in cirrhosis leading to overproduction of proinflammatory cytokines, which has been seen both in vivo 43 and ex vivo. 44 Additionally, owing to a persistent passage of PAMPs from a dysfunctional intestinal barrier, 45 46 PRRs such as TLR-4 and Caspases-4/5 are upregulated in the parenchymal cell of the liver, kidneys, and the brain. 47 48 Effectively, this produces a chronic state of low-grade systemic inflammation, which over time leads to loss of tolerance to antigen recognition with augmented proinflammatory response. 49 Inactivation or removal of PAMPs from the gut therefore is protective against the development of this chronic inflammatory state and ACLF.

Addressing the increased gut permeability in the acute setting is challenging as factors leading to this are multiple. Moreover, permeability itself is worsened by dysbiosis, thus creating a vicious cycle. 50 Therefore, gut decontamination has been one of the first explored mechanisms to address this issue. Here, nonabsorbable antibiotics such as rifaximin or norfloxacin were shown to reduce the severity of endotoxinemia 51 52 and blunt the hyperdynamic circulatory state, 52 thereby preventing the recurrence of hepatic encephalopathy (HE), 51 spontaneous bacterial peritonitis (SBP), and all-cause decompensation, thus improving survival. 32 53

Furthermore, nonantibiotic gut decontaminating agents, such as CARBALIVE and recombinant alkaline phosphatase (recAP), follow the same principle. CARBALIVE is a novel engineered macroporous carbon bead, which when ingested orally is capable of binding toxins and cytokines of molecular weights of up to 70 kDa. 54 While shown to be well tolerated, CARBALIVE dampens systemic inflammation and improves gut permeability. 55 Elsewhere, the observation that alkaline phosphatase, a marker of cholestasis, possesses anti-inflammatory activity through the dephosphorylation of LPS, and thus, its deactivation led to the development of recAP. 56 Thereafter, treatment with recAP in an animal model of ACLF was shown to reduce liver injury, inflammation, and cell death through downregulation of TLR-4 expression. 57 Interestingly in the same study a similar effect was not observed in the acute liver failure (ALF) model, which may point to the fact that recAP exerts its effect through LPS rather than PPRs. 57

Restoration of gut microbiome using fecal microbial transplant (FMT) has the potential to reduce translocation thus limiting the passage of PAMPs into systemic circulation. 22 While investigations into the safety and efficacy of this are ongoing, small-scale studies have shown a benefit in the prevention 58 and treatment of HE 59 in patients with decompensated cirrhosis (DC). One important aspect of FMT safety is the rigorous adherence to the donor screening guidelines as infections have been reported in immunosuppressed patients including in cirrhosis. 60 Finally, while it seems inconceivable for FMT to be used in AD or ACLF at this current point in time, it may have an important role in prevention of relapse in patients recovering from the acute injury.

### Targeting Damage-Associated Molecular Patterns

Damage-associated molecular patterns (DAMPs) are the products of necrotic cells. Here again the generation of inflammatory response is mediated by specific PRRs, which under normal circumstances would carry out the end goal of promoting tissue repair. 61 Accumulating evidence suggests that nonapoptotic cell death (necroptosis and pyroptosis) is the dominant form of cell death in ACLF. 23 62 This is more immunogenic than the apoptotic form. 23 63 64 65 A subset analysis of patients from the CANONIC study 66 showed that the level of circulating caspase-cleaved keratin 18 (cK18) and keratin 18 (K18), representing apoptotic and total cell death, respectively, increased with severity of the syndrome. Interestingly, the apoptotic index (cK18:K18 ratio) decreased as the clinical severity increased in patients with ACLF, suggesting perhaps that, while both apoptotic and necrotic cell deaths increase with increasing severity of ACLF, the necrotic forms predominate in severe ACLF.

The evolution from apoptosis to nonapoptotic form of cell death with worsening severity of ACLF may partly explain the limited effect of emricasan, a pan-caspase inhibitor, on the treatment of ACLF. 67 This is an oral pan-caspase protease inhibitor, which has been studied in patients with cirrhosis who presented with acute deterioration of liver function and associated organ failure. 64 While a sufficient effect on caspase 3/7 activity was observed, this was not associated with any significant change in disease severity after 7 days. Future trials of emricasan may need to exclude those with ACLF as highlighted by its success in reducing the Model for End-Stage Liver Disease (MELD) score in patients who had Child–Pugh scores of A and B at baseline. 68

Necroptosis results from engagement of the receptor-interacting protein kinases (RIPK) 1 and 3 65 leading to the formation of necrosomes. This then brings about cell swelling and release of DAMPs, driving further inflammation and organ failures. 62 A study by Kondo et al 23 revealed that rising plasma RIPK3 concentration was associated with the progression and severity of ACLF, highlighting the importance of necroptosis in ACLF. Interestingly, RIPK3 levels were significantly higher in those with alcohol-related cirrhosis and those with infection especially in the presence of organ failure (except respiratory) with the highest levels in those with multiorgan failure. Moreover, the levels of RIPK3 correlated with that of WBC and other markers of inflammation (IL-6, IL-8, IL1ra, and sCD163) as well as markers of cell death (K18 and cK18). The authors then in a rodent model of ACLF observed that inhibition of necroptosis by necrostatin-1 (NEC-1) and SML2100 (RIPA56) prevented the occurrence of ACLF. 23 Pyroptosis results from noncanonical inflammasome activation. 69 Here, caspases-4/5 in humans, and caspase-11 in mice, act as PRR for intracellular LPS (and other PAMPs) and subsequently cleave the effector protein Gasdermin-D leading to pore formation in the plasma membrane. 70 This precipitates the lytic form of cell death and release of more DAMPs, worsening systemic inflammation. 67 A study by Soffientini et al demonstrated a link between pyroptosis, hepatic and extrahepatic organ dysfunction in animal models and primary human hepatocyte models. Here, *Casp11* ^−^ / ^−^ mice with advanced liver fibrosis were protected from hepatocyte and multiorgan injury compared with the wild type. 48 Hu et al 71 recently demonstrated that Disulfiram is an effective inhibitor of Gasdermin-D aggregation and may consequently be repurposed to inhibit pyroptosis. A Phase IIa trial of Disulfiram in high-risk AD and grade 1 ACLF is in progress. Therefore, inhibition of both the necroptotic and pyroptotic cell death pathways may be potential novel approaches.

### Targeting Albumin Dysfunction

**Fig. 2:**
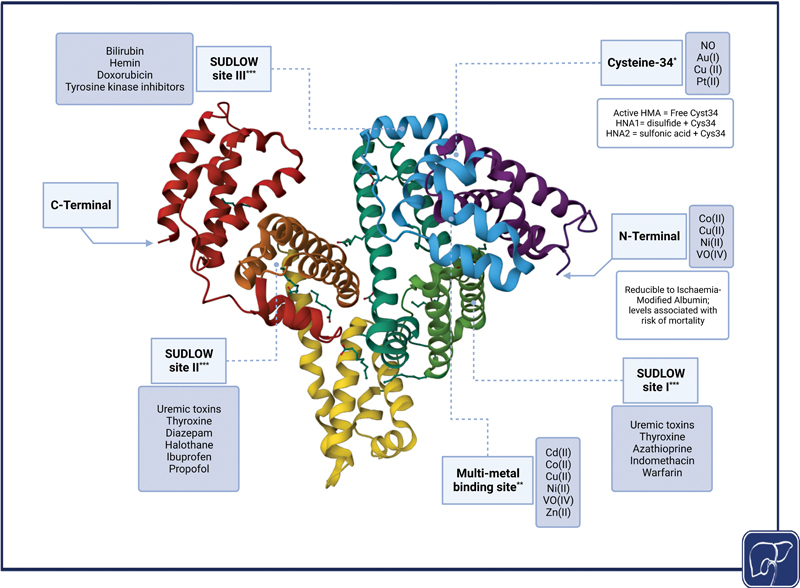
Structure of albumin with its binding sites and the main compounds that bind to each one. * Cyst34 state can be assessed using HPLC and its inactivation is associated with mortality. ** Multimetal binding site can be assessed using cobalt-binding assay. *** SUDLOW binding sites can be assessed using electron paramagnetic resonance spectroscopy and their loss of function is associated with mortality. Albumin structure was modified and downloaded from RCSB PDB ( https://doi.org/10.2210/pdb1E78/pdb ) of PDB ID 1E78. 147 ACLF, acute-on-chronic liver failure; HPLC, high performance liquid chromatography; RCSB PDB, Research Collaboratory for Structural Bioinformatics Protein Data Bank.

Human serum albumin is the most abundant plasma protein, synthesized exclusively by the liver, and is a major contributor to plasma oncotic pressure. Additionally, it is a pleiotropic scavenger, reversibly binding to a plethora of molecules including toxic metabolites, reactive oxygen species, and inflammatory mediators 72 ( Fig. 2 ). The structural integrity and function of albumin can become severely deranged in the presence of systemic inflammation and oxidative stress, leading to many posttranslational changes such as conversion of active human mercaptalbumin (HMA) reversibly or irreversibly, to human nonmercaptalbumin-1 (HNA-1) or HNA-2, respectively. 73 These changes in the structure and function of albumin directly correlate with the severity of cirrhosis and that of systemic inflammation. 74 75 Therefore, the proportion of albumin possessing full function, referred to as “effective albumin concentration,” is much lower than the routinely measured serum concentration levels. 76

The guidelines-based therapeutic roles of albumin are in the prevention and treatment of circulatory dysfunction and renal failure associated with large volume paracentesis and SBP and in the treatment of type-1 hepatorenal syndrome. Its role in treatment of AD and ACLF, and in their prevention, is a subject of intense investigation.

With regard to its capacity in treatment of patients with AD or ACLF, there are a few studies, which report benefit. The DIALIVE study, which is discussed later, is an example of this. 77 Similarly, a pilot study of the ongoing APACHE trial ( Table 2 ) performed by the study sponsor in 10 patients with ACLF, showed improved liver, renal, cardiovascular, and cerebral function and attenuation of inflammatory response and endothelial dysfunction. Serum albumin concentration increased from 24 to 28 g/L. Short-term mortality rate was 30% at 28 days and 40% at 90 days. These mortality rates were lower than those observed in 40 matched subjects of ACLF in the CANONIC Study (57.5 and 65%, respectively). Currently running, the APACHE study (NCT03702920) involves plasma exchange with 5% human albumin solution (HAS) to treat patients with ACLF grades 1b to 3a. This is done across 4 to 9 sessions, depending on the treatment response, over a 17-day period. This strategy combines the benefits of replacing ineffective albumin with the removal of plasma inducers of inflammation.

**Table 2:** Summary of ongoing clinical trials

Trial number/name	Study design/phase	Study objectives	Target population	Scientific rationale
NCT03202498 (CARBALIVE)	Randomized, safety trial, multicenter study	To evaluate safety and tolerability of CARBALIVE in patients with DC	28 patients	Synthetic carbon has a high absorption capacity for bacterial products as well as proinflammatory cytokines
NCT04229901 (HALPC)	Phase IIb randomized, placebo-controlled, double-blind, multicenter study	To evaluate efficacy and safety of HepaStem (human allogeneic liver-derived progenitor cells [HALPC] therapy) in patients with ACLF	363 participants	HALPC have a liver-specific homing capacity after peripheral intravenous infusion in addition to its immunomodulatory and antifibrotic properties
NCT05030571	Open-label, randomized controlled, single-center study	To compare the clinical and immunomodulatory effects of double plasma molecular adsorption system (DPMAS) to standard of care in ACLF patients	40 participants	Plasma filtration and toxins removal to improve hepatocyte function and regeneration
NCT04597164	Multicenter, prospective cohort study	To evaluate the safety and efficacy of combination therapy with DPMAS and low volume plasma exchange vs standard medical treatment in patients with HBV related ACLF	200 participants	Plasma filtration and toxins removal to improve hepatocyte and regeneration
NCT05019352 (CYTOHEP)	Single-center, open-label, randomized, controlled intervention trial	To assess the benefit of extracorporeal hemoadsorption using the CytoSorb device in patients with ACLF	51 participants	Reduction of serum bilirubin in critically ill patients to allow recovery in ACLF based on previous proven benefits in ALF patients
NCT04431375	Open-label, randomized, controlled intervention trial	To study the efficacy of addition of fecal microbiota transplant (FMT) and plasma exchange to tenofovir compared with monotherapy with tenofovir in patients with HBV reactivation who develops ACLF	70 participants	Restoration of the normal gut microenvironment with subsequent improvement of gut liver axis dysfunction
NCT03451292 (PRECIOSA)	Phase 3 Multicenter open-label, randomized, controlled intervention trial	To evaluate the efficacy of long-term albumin 20% administration vs. standard medical treatment in subjects with DC and ascites	410 participants	Improving the hemodynamic dysfunction in patients with DC. Immunomodulatory and detoxifying properties of albumin
NCT03702920 (APACHE)	Phase 3 Multicenter, randomized, controlled, parallel group, open-label study	To evaluate the effects of plasma exchange using human serum albumin 5% in ACLF patients	380 participants	Removal of the circulating toxins to provide a suitable environment for suppression of systemic inflammation and ACLF recovery

Abbreviations: ACLF, acute-on-chronic liver failure; DC, decompensated cirrhosis; HBV, hepatitis B virus.

On the other hand, results of studies assessing the preventative role of albumin in clinical trials have shown mixed results. Except the ANSWER (Albumin for the Treatment of Ascites in Patients with Hepatic Cirrhosis) study, none of these have shown significant benefit. In the ANSWER study, 78 performed in patients with persistent ascites, favorable results were observed with albumin therapy, with a 38% reduction in the hazard ratio for mortality. Furthermore, there was a 50% reduction in the incidence of refractory ascites, as well as reduction in the incidence of complications of cirrhosis including risk of renal dysfunction, HE, and risk of SBP and non-SBP bacterial infections resulting in significantly fewer liver-related admissions to hospital. Conversely, the MACHT (Midodrine and Albumin for Cirrhotic Patients in the Waiting List for Liver Transplantation) study 79 did not reproduce similar outcomes. It must be noted that the MACHT study comprised patients with DC awaiting liver transplant with a higher average MELD score compared with the ANSWER study. Furthermore, in the MACHT study the regime included midodrine (15–30 mg/d), an α-1 receptor agonist, yet more importantly the dosing of albumin significantly differed between the two studies.

Lastly, the ATTIRE (Albumin to Prevent Infection in Chronic Liver Failure) study, is a large randomized controlled trial (RCT) where HAS was given as an infusion (rate = 100 mL/h, for maximum of 14 days) with the aim of maintaining an albumin level of 35 g/L or less. 80 This strategy was based on a previous finding that an albumin level of 30 g/L or less was predictive of immune dysfunction in patients with cirrhosis. 81 Despite administering a median dose of 200 g (interquartile range [IQR]: 140–280) in the treatment group versus 20 g (IQR: 0–120) in the control group, the study failed to show a significant difference in any of its primary or secondary outcomes. In fact, albumin infusion was associated with a higher rate of pulmonary edema.

When considering the results of these studies, we must first note that the results of the ANSWER study were confirmed in the pilot-PRECIOSA and INFECIR-2 studies. Here, patients with stable DC were shown have an improvement in the systemic inflammation only when given high-dose albumin therapy (defined as 1.5 g/kg/wk). 82 Therefore, it appears that achieving serum albumin levels like that of healthy individuals (∼ 40 g/L) makes physiological sense to derive maximum benefit from albumin therapy in liver disease. However, in the case of the MACHT study the patients were significantly short of this mark, whereas the brief treatment period in the ATTIRE study could have failed to establish a lasting effect, while potentially increasing the risk of fluid-overload. Finally, it is worth highlighting that even in the commercially available HAS only around 50% of the albumin is in the active HMA form. 83 Therefore, it is of paramount importance for future studies to assess the change in the effective albumin concentration rather than the numerical serum levels.

Currently, the PRECIOSA study (NCT03451292) is exploring the role of albumin to better identify patients who can benefit from long-term HAS replacement and determine a criterion for dosing and frequency of treatment. Here, the study population includes patients with AD of cirrhosis with or without ACLF to whom 20% HAS is given at a dose of 1.5 g/kg (maximum 100 g/session) every 8 to 12 days over a 1-year period.

### Targeting Ammonia

In ACLF patients with HE, increased ammonia levels have been shown to be associated with longer intensive care unit (ICU) stays and the failure to improve levels with significant mortality risk. 84 85 An ammonia level greater than 79.5 µmol/L has been shown to be significantly associated with a higher grade of HE as well as a higher risk for all other organ failures. 86 Moreover, studies have shown that hyperammonemia impairs neutrophil function by reducing chemotaxis 87 and phagocytosis while increasing spontaneous oxidative burst 24 with the latter being associated with increased risk of infection and mortality. 88 Hyperammonemia also plays a role in the development of sarcopenia through upregulation of myostatin expression that negatively impacts skeletal mass. 89 It is therefore not surprising to see that a high ammonia level (≥ 79.5 µmol/L) is associated with a higher frequency of organ failures and mortality. 86 While the kidneys can adapt to high levels in the earlier stages of the disease, the capacity for ammonia clearance diminishes with worsening acidosis and hypokalemia. 90

The association of hyperammonemia in the increased risk of infection and development of organ failures highlights the importance of ammonia-lowering strategies. Currently, approved therapies are limited to lactulose and rifaximin with good evidence for their effect in prevention of HE. 91 While rifaximin has been shown to have a positive impact on survival in patients with DC, 92 93 the effect of lactulose alone on survival outside of patients with HE is unclear. 94 Elsewhere, L-ornithine phenylacetate (OP) has been shown to improve ammonia levels in patients with cirrhosis 95 with its safety confirmed. 96 In a rodent model of ACLF, OP was shown to prevent LPS-driven coma and reduce brain water content while reducing inflammatory cytokine (TNFα and IL-6) and ammonia levels. 97 The results of a phase-2 study of OP in patients with cirrhosis and HE showed a shorter time to clinical improvement when compared with standard medical therapy (SMT, which included lactulose and rifaximin) leading to a numerical advantage in time to discharge from ICU. 98

Another innovative approach to address hyperammonemia is through injection of particles capable of adsorbing ammonia. VS-01 achieves this by use of large transmembrane pH-gradient liposomes containing citric acid. This is delivered into the peritoneal cavity via paracentesis. Once inside the peritoneum, uncharged ammonia diffuses out of the blood stream into the peritoneal cavity and across the liposomal bilayer membrane where it becomes trapped due to its positive charge. Interestingly, many other toxins that are present in excess, e.g., urea, can be removed via the same mechanism. At the end, these ammonia-containing liposomes are removed as ascitic fluid is drained. 99 First human trial of VS-01 reported its safety while serum ammonia levels were successfully reduced (abstract only). 100

### Targeting Toll-like Receptor 4 and Inflammasome Pathways

**Fig. 3:**
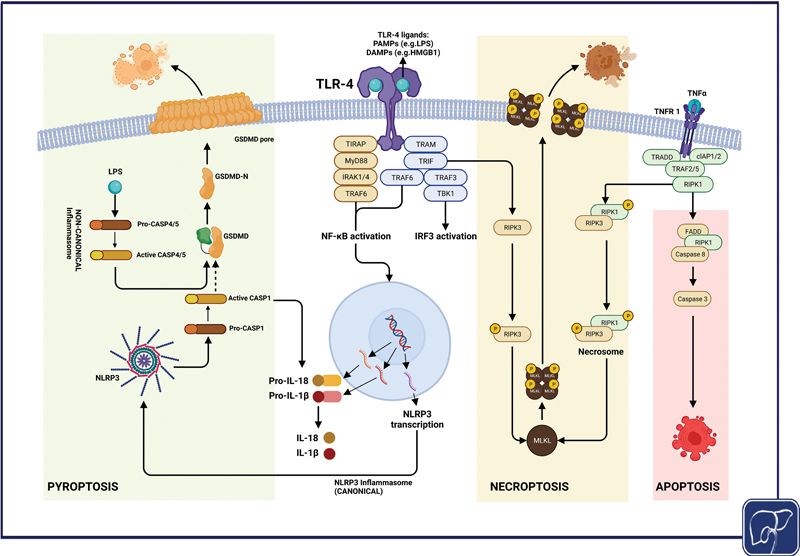
Overview of modes of cell death in cirrhotic liver disease. Necroptosis and pyroptosis are the dominant forms of cell death in ACLF and TLR-4 and its downstream pathways play a key role in their activation. Intracellular LPS activates noncanonical inflammasomes resulting in CASP4/5 formation. Inactive GSDMD protein is then cleaved into its active form, GSDMD-N, which forms pores in the plasma membrane leading to pyroptotic cell death. Additionally, noncanonical inflammasomes activate NLRP3 (not shown in the figure) resulting in cleavage of pro-IL-1β and pro-IL-18 into their active forms. Formation of necrosome complex can be initiated by a variety of factors of which the activation of TNF receptor remains the principal trigger. TLR-4 activation can also directly activate RIPK3 thus triggering MLKL and pore formation in the cell membrane. The result of necroptotic or pyroptotic cell death is the release of cellular contents including DAMPs, cytokines, and chemokines, which further worsen the state of systemic inflammation. ACLF, acute-on-chronic liver failure; ASC, apoptosis-associated speck-like protein containing a CARD; CARD, caspase recruitment domain; CASP1, caspase-1; CASP4/5, caspase-4 or 5; DAMP, damage-associated molecular pattern; GSDMD, gasdermin D; IL, interleukin; LPS, lipopolysaccharide; MLKL, mixed linage kinase domain-like; NF-κB, nuclear factor κB; NLR, nucleotide-binding domain; NLRP, NOD-like receptor family; PYD, pyrin domain; RIPK, receptor-interacting protein kinase; TNF-α, tumor necrosis factor α; TLR-4, toll-like receptor-4.

The role of TLR-4 receptor in mediation of systemic inflammation has been highlighted many times as evidenced by its upregulation 47 101 and increased concentration of its ligands 102 in patients with cirrhosis. TLR-4 is present on parenchymal and nonparenchymal cells such as hepatocytes and hepatic stellate cells where its activation results in increased proinflammatory cytokine production, 20 caspase activation, and cell death ( Fig. 3 ), 103 as well as senescence through production of factors such as transforming growth factor-1β. 104 Inhibition of TLR-4, whether through removal of its ligands by gut decontamination 105 or through the direct inhibition of the receptor itself 102 has been shown to improve survival in animal models. TAK-242 is a small molecule that selectively binds and inhibits TLR-4. Strangely, its administration in human ovarian cancer cell lines was seen to result in cell cycle arrest preventing proliferation. 106 Therefore, in a second animal study TAK-242 was combined with granulocyte–colony stimulating factor (G-CSF) 104 (the effect of which in ACLF will be discussed further on) creating a combinatorial therapy called G-TAK, which proved to be effective in preventing inflammation and senescence thus inducing regeneration. This effect was validated in two different animal models of ACLF. Hence, the safety and efficacy of G-TAK will be put test for the first time in man in a multicenter European study called A-TANGO (NCT04620148), which is set to commence in 2023.

### Targeting Hepatic Regeneration

Owing to its anatomical position and its role in rendering toxins harmless, the liver is under the constant threat of injury. Hence, it possesses a robust capacity for renewal. Here exists a three-tier system: (1) hepatocyte-mediated regeneration, (2) hepatic progenitor cell (HPC)-mediated regeneration with capacity for hepatocyte and nonhepatocyte cell renewal, 107 and (3) hemopoietic cell mobilization from the bone marrow that can contribute to hepatic parenchymal and nonparenchymal cell regeneration. Under normal circumstances, hepatocyte turnover is slow, yet after acute injury (e.g., partial hepatectomy), rapid mitotic restoration can occur within two division cycles. However, a different set of dynamics govern ACLF. Here the HPC-mediated pathway becomes dominant and hepatocyte proliferation abrogated, demonstrated by the decrease in Ki67 positive cells (marker of hepatocyte-proliferation) and an increase in the cytokeratin 19 positive cells (marker of HPCs). Additionally, ACLF results in a significant increase in ductular reaction, which is also indicative of HPC-mediated regeneration. More importantly, this reduction in hepatocyte proliferation is significantly associated with a poor outcome where the number of Ki67 positive cells is predictive of mortality. 26

The discovery of bone marrow participation in hepatic regeneration has led to the use of G-CSF in patients with ACLF as it induces hematopoietic stem cell mobilization to the peripheral blood with an additional neutrophil-activating effect, which considering the immunoparalytic state of ACLF, makes it attractive. Certainly, the early trials confirmed this positive impact. In 2012 Garg et al 108 published their results of treating patients with ACLF (defined by the APASL criteria) with 5 µg/kg of G-CSF subcutaneously over a 1-month period in comparison with SMT. A significant improvement in the lymphohemopoietic CD34+ stem cells was seen, which was associated with fewer infections and an improved 60-day mortality (69 vs. 29%, *p* = 0.01). Similar positive results were reproduced in patients with severe alcoholic hepatitis 109 and in those with DC without ACLF. 110 However, two large multicenter European studies have cast doubt on the earlier optimistic views. First was the REALISTIC study, 111 conducted in patients with compensated cirrhosis, and second was the GRAFT study 112 in patients with ACLF (according to the EASL-CLIF criteria). Neither of these studies showed benefit toward survival compared with SMT. The rate of infection was also unaffected by G-CSF therapy in the GRAFT study. Furthermore, while the authors of the GRAFT study used a different protocol to that of Garg et al; their subanalyses in the patients who fulfilled the APASL ACLF criteria did not show a significant change in survival. At the same time seven drug-related serious adverse events were reported. G-CSF, alone, is therefore not recommended for use outside clinical trials.

The failure of G-CSF therapy alone points to its ability to act as a double-edged sword. While on one hand the mobilization of stem cells is deemed beneficial, the effect on monocytes and macrophages can be proinflammatory or worsen sepsis. 113 Furthermore, the bone marrow mobilization capacity in cirrhosis is thought to be reduced, 111 yet the exact degree of this and its implications on G-CSF therapy remain unexplored.

Cell therapy is another approach for the treatment of regenerative failure seen in ACLF. Here mesenchymal stem cells (MSC) can be isolated from bone marrow, other adult tissues (e.g., adipose tissue), or embryonic sources (e.g., umbilical cord). One issue with these methods is the limited availability of the appropriate tissues. Therefore, the use of induced pluripotent stem cells has emerged as an alternative. 114 While detailed review of these methods is beyond the scope of this article and has been discussed elsewhere, 115 it is worth noting that two single-center studies in China 116 117 have shown survival benefit from injection of MSCs in patients with ACLF (with hepatitis B etiology). Further interpretation of the results is difficult due to the heterogeneity in cell sourcing, administration, as well as the study populations. Subsequently, two RCTs 111 118 were performed where bone marrow-derived MSCs was combined with G-CSF, one in patients with decompensated alcoholic liver disease and the other in those with compensated cirrhosis (randomized against treatment with SMT or G-CSF alone, respectively). Again, the mode of stem cell administration differed between the two studies as did the treatment regimen. Regardless, both studies did not show significant clinical improvement. This lack of effect may be attributed to insufficient engraftment which potentially could be addressed by dose alterations. Yet it is difficult to comment on this further due to the limitations in cell tracing techniques as concerns exists regarding the effect cell labeling could have on their viability.

Another novel approach to the idea of multipotent cell therapy is with human allogenic liver-derived progenitor cells (HALPC), which is currently under investigation in a multicenter RCT (NCT04229901). Here the progenitor cells are cultured from the progenitor fraction of healthy human liver tissue. The results of the phase-2 study in patients with alcoholic hepatitis showed that HALPC is safe while gradually improving markers of systemic inflammation and liver injury over a 3-month period. 119

### Targeting Endothelial Dysfunction

Endothelial dysfunction is an important feature of cirrhosis, one that is highlighted by an impaired vasodilator response to acetylcholine, reduced production of nitric oxide (NO) by endothelial nitric oxide synthase (eNOS), as well as increased NO breakdown due to inflammation and oxidative stress. 120 121 This then leads to impaired endothelial relaxation in the hepatic microcirculation contributing to increased intrahepatic vascular resistance. 122 On the other hand, in the splanchnic vessels, overproduction of NO leads to vasodilation and hyperdynamic vascular disturbances. 18

Evidence shows that in ACLF there is a significant decrease in plasma stromal cell derived factor-1 levels, a marker of reduced liver endothelial and mesenchymal/stromal cell regenerative activity, compared with patients with ALF. 26 Another important observation is that inhibition of HMG-CoA reductase can result in increased NO bioavailability through inhibition of the Rho-ROCK pathway highlighting the potential role of statins to treat endothelial dysfunction. Statins also have anti-inflammatory action and are known to block the action of eNOS downregulators such as the oxidized low-density lipoprotein, TNF-α, and caveolin-1. Lastly, statins upregulate the activity of antioxidant enzymes catalase and paraoxonase. 120

A randomized blinded trial of simvastatin versus placebo showed that after 3-month period those treated were significantly more likely to have a decrease in their hepatic venous pressure gradient. Notably, the study also reported a greater benefit in those with medium to large esophageal varices with previous history of bleeding. 123 Such findings led to creation of the Liverhope study (NCT03150459). In this study, rifaximin 400 mg, a poorly absorbed antibiotic, is combined with simvastatin 20 mg. While rare, statins can result in acute liver injury. 124 In the Liverhope-Safety study the safety of simvastatin at a dose of 40mg/d came under question due to a significantly increased risk of adverse events such as rhabdomyolysis. 125 Conceivably, this risk may be more exaggerated in patients with ACLF and therefore requires careful evaluation. Regardless, the results of the Liverhope study failed to show any clinical benefit compared with placebo in the occurrence of ACLF in susceptible population (Oral presentation at EASL 2023, Vienna, LBO-01).

### Targeting Mitochondrial Dysfunction

Significant metabolic shifts have been observed in DC and ACLF. The high-throughput blood metabolomics analyzed in the CANONIC cohort revealed that mitochondrial dysfunction contributes to defective cellular energy production, resulting in the development of peripheral organ failure. 27 Currently, there are no specific treatments being explored targeting mitochondrial dysfunction in ACLF.

### Targeting Organ Immunopathology and Organ Support

There are two broad categories of liver support devices: artificial (mechanical) and bioartificial (cell-based devices). The best studied artificial devices are those that rely on the principles of albumin dialysis. Molecular Adsorbent Recirculation System (MARS) utilizes HAS as dialysate allowing for a more effective uptake of protein-bound toxins (as well as water-soluble toxins) across a polysulfone membrane. Here, HAS is then recycled for further rounds of detoxification. 126 This is the main advantage MARS has over a single pass albumin dialysis system, where HAS is discarded after single use, resulting in far greater volumes to be required. 127 An early and small group trial of MARS in patients with alcohol-related ACLF 128 resulted in improvement in bilirubin and bile acid levels as well as grade of HE and Child–Pugh scores. Yet, the RELIEF study failed to show benefit in the overall survival rate. 129 However, an increase in the dosage and duration of treatment was retrospectively explored in a meta-analysis of individual patient data from three RCTs in ACLF patients treated with MARS. 130 This confirmed high-intensity therapy (≥ 5 sessions) to be associated with a better survival rate. Prometheus system uses fractionated plasma separation and adsorption through a membrane with a greater permeability cutoff (250 vs. 50 kDa in MARS), thus allowing albumin to cross through. 131 The HELIOS trial failed to show a significant change in the 28- or 90-day transplant-free survival rates. 132 Neither of these approaches are recommended for routine use.

Biological devices focus on replacing the lost synthetic function of the liver. These devices are comprised of a bioreactor and a cellular component. The bioreactor is where the patient's plasma is separated through a membrane, before being perfused through the cellular component. Due to limited accessibility to human hepatocytes, the cellular component is often porcine in origin. Alternatively, genetically engineered hepatocytes such as C3A, HepG2, and HHY41 immortalized human cell lines can be used. The extracorporeal liver-assist device utilizes 200 g of human hepatoblastoma C3A cell line in its cellular component. It underwent large clinical trials in patients with severe alcoholic hepatitis, many of whom had ACLF but no survival benefit was observed. 133

DIALIVE, a novel device that has been built to specifically address the pathophysiological derangements responsible for the development of ACLF and avoid one of the reasons underlying the possible failure of previously used devices. 134 DIALIVE incorporates a renal dialysis machine and uses a dual-filtration system connected in series. The first filter is comprised of a membrane that allows ultrafiltration of albumin and cytokines, and the second filter adsorbs PAMPs and DAMP. The removed albumin is replaced in similar quantities with bottled, 20% HAS. Major differences of DIALIVE system compared with MARS and Prometheus are that albumin removed is not recirculated in DIALIVE with wasted albumin replaced by bottled albumin, and the additional endotoxin filter in the DIALIVE system addresses endotoxemia by removing (adsorbing) endotoxins. An RCT of DIALIVE versus SMT has been recently performed in 32 patients with ACLF. DIALIVE was shown to be safe with no differences in adverse events between the two groups. A significant reduction in the severity of endotoxemia and improvement in albumin function was observed in the DIALIVE group, which translated into a significant reduction in the CLIF-C organ failure and CLIF-C ACLF scores at Day 10 and a faster time to resolution of ACLF. 77 Biomarkers of systemic inflammation, cell death, endothelial function, and ligands for TLR-4 and inflammasome improved significantly in DIALIVE group. These data including evidence of clinical and pathophysiological effects of DIALIVE provide compelling rationale to proceed to registration clinical trials.

### Liver Transplantation

**Fig. 4:**
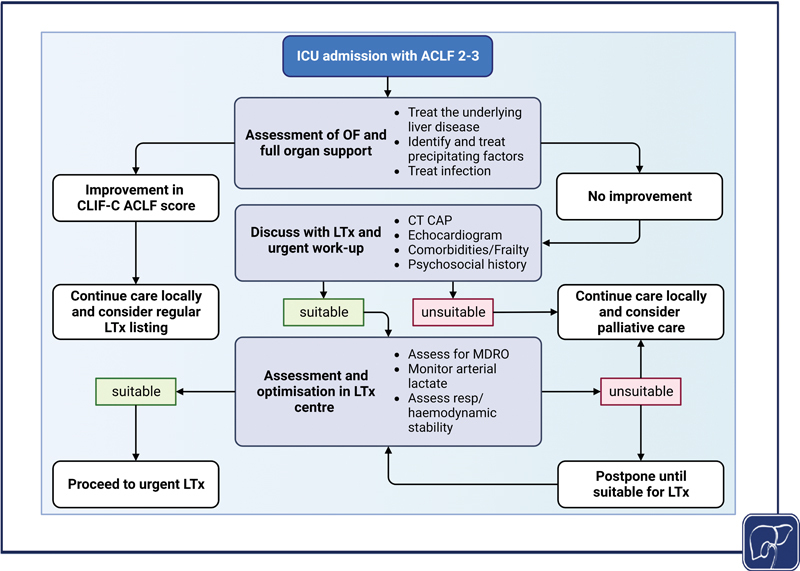
Suggested treatment algorithm for treatment of patients with ACLF grades 2 to 3. All four graphical figures in this review article were created with BioRender.com. ACLF, acute-on-chronic liver failure; CT CAP, computed tomography scan of chest, abdomen, and pelvis; LTx, liver transplantation; OF, organ failure; MDRO, multidrug-resistant organism.

LTx is currently the only rescue option in ACLF patients unresponsive to standard care. This has been shown to improve outcome in patients with ACLF with 1-year survival exceeding 80% regardless of ACLF grade. 14 15 16 It should be highlighted that the long-term survival rate of patients with ACLF after LTx is similar to those with no ACLF. 17 Nevertheless, several barriers exist in terms of prioritization, organ allocation, and post-LTx survival optimization among this population. Although the survival benefit of LTx in ACLF patients was clearly demonstrated before, most of these studies were retrospective, conducted on small number of patients, with no standardization of ACLF definition or transplant prioritization criteria 135 136 137 138 ( Table 3 ). These gaps are currently being addressed in the ongoing global CHANCE (NCT04613921) study to standardize the liver transplant practice in ACLF patients. Nevertheless, LTx is the only treatment that prolongs survival and all patients with ACLF should be considered. An algorithm describing management of ACLF patients, assessment for transplantation, and futility is described in Fig. 4 .

**Table 3:** Summary of selected studies describing outcome of liver transplantation in patients with acute-on-chronic liver failure

Author/reference	Population number	Number with ACLF grade 3	Country of origin	Definition	1-year mortality post-LTx in ACLF grade 3 (%)
Levesque et al 135	140	30	France	CLIF	56.7
Artru et al 136	337	73	France	CLIF	16.1
Bhatti et al 139	60	2	Pakistan	CLIF	0 at 3 mo
Sundaram et al 137	21,269	6,381	United States	CLIF	18.2
Marciano et al 140	60	8	Argentina	CLIF	17.5
Agbim et al 138	101	191	United States	CLIF	17.5
Artzner et al 141	152	152	4 centers in France, 1 center in the United Kingdom	CLIF	26
Belli et al 142	234	98	8 European countries	CLIF	21.1
Xia et al 143	162	47	China	CLIF	30.2 at 3 y
Artzner et al 144	98	98	8 European countries	CLIF	21
Finkenstedt et al 145	238	144 had ACLF	Austria	APASL	18 for all ACLF
O'Leary et al 146	2,793	119	United States	NACSELD	7 for all ACLF at 6 mo

Abbreviations: ACLF, acute-on-chronic liver failure; APASL, Asian Pacific Association for the Study of the Liver; CLIF, Chronic Liver Failure Consortium; LTx, liver transplantation; NACSELD, North American Consortium for the Study of End-stage Liver Disease.

Source: Reproduced from Lee et al 2015. 134

## Conclusions and Future Perspectives

ACLF is a complex clinical condition with inherent heterogeneity in the underlying chronic liver disease, diverse precipitating insults and multiplicity of pathogenic processes driving systemic inflammation, immune dysfunction, and cell death, which collectively result in various combinations of end-organ damage. Our understanding of the disease has improved greatly over the recent years and great strides made toward finding novel therapeutic approaches and interventions most of which are still preclinical and in animal models. Several clinical trials are underway that will provide greater insight into treating this disease condition that carries a huge mortality rate ( Table 2 ).

## Notes

Lay Summary Acute-on-chronic liver failure (ACLF) occurs in patients with scarring of the liver, who sustain a further injury. This condition carries a high mortality rate with no definitive treatments beyond liver transplantation. Here, we discuss the mechanisms that govern ACLF, which can be targeted for treatment. We also describe the clinical trials that aim to address this unmet need.
